# Biochemical Properties of Tyrosinase from *Aspergillus terreus* and *Penicillium copticola*; Undecanoic Acid from *Aspergillus flavus,* an Endophyte of *Moringa oleifera,* Is a Novel Potent Tyrosinase Inhibitor

**DOI:** 10.3390/molecules26051309

**Published:** 2021-03-01

**Authors:** Hanaa Salah Maamoun, Gamal H. Rabie, Ibrahim Shaker, Bothaina A. Alaidaroos, Ashraf S. A. El-Sayed

**Affiliations:** 1 Enzymology and Fungal Biotechnology Lab., Botany and Microbiology Department, Faculty of Science, Zagazig University, Zagazig 44519, Egypt; micro_hh@yahoo.com (H.S.M.); rabigamal@yahoo.com (G.H.R.); 2 Limnology Department, Central Laboratory of Aquaculture Research, Tell El Kebir 41626, Egypt; sabry@yahoo.com; 3 Biology Department, Faculty of Science, King Abdulaziz University, Jeddah 21955, Saudi Arabia; Bothainaad@gmail.com

**Keywords:** *Aspergillus terreus*, *Penicillium copticola*, tyrosinase, kojic acid, undecanoic acid

## Abstract

Tyrosinase is a copper-containing monooxygenase catalyzing the *O*-hydroxylation of tyrosine to 3,4-dihydroxyphenylalanine then to dopaquinone that is profoundly involved in melanin synthesis in eukaryotes. Overactivation of tyrosinase is correlated with hyperpigmentation that is metabolically correlated with severe pathological disorders, so, inhibition of this enzyme is the most effective approach in controlling the overproduction of melanin and its hazardous effects. Thus, searching for a powerful, selective inhibitor of human tyrosinase to limit the hyper-synthesis of melanin is a challenge. Unlike the difficulty of overexpression of human tyrosinase, using fungal tyrosinase as a model enzyme to the human one to evaluate the mechanistics of enzyme inhibition in response to various compounds is the most feasible strategy. Thus, the purification of highly catalytic-efficient fungal tyrosinase, exploring a novel inhibitor, and evaluating the mechanistics of enzyme inhibition are the main objectives of this work. *Aspergillus terreus* and *Penicillium copticola* were reported as the most potential tyrosinase producers. The biochemical properties suggest that this enzyme displays a higher structural and catalytic proximity to human tyrosinase. Upon nutritional bioprocessing by Plackett–Burman design, the yield of tyrosinase was increased by about 7.5-folds, compared to the control. The purified tyrosinase was strongly inhibited by kojic acid and *A. flavus* DCM extracts with IC_50_ values of 15.1 and 12.6 µg/mL, respectively. From the spectroscopic analysis, the main anti-tyrosinase compounds of *A. flavus* extract was resolved, and verified as undecanoic acid. Further studies are ongoing to unravel the in vivo effect and cytotoxicity of this compound in fungi and human, that could be a novel drug to various diseases associated with hyperpigmentation by melanin.

## 1. Introduction

**Scheme 1:**
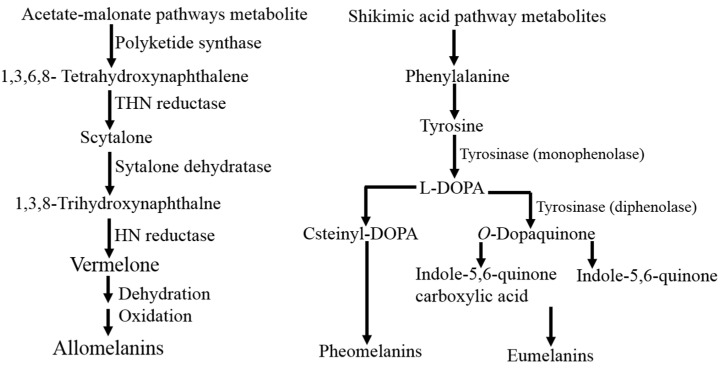
Biosynthesis of melanin in human and fungi.

Tyrosinase is a copper-containing monooxygenase (EC 1.14.18.1) catalyzing the *O*-hydroxylation of monophenols into *O*-diphenols (monophenolase) and oxidation of *O*-diphenols to *O*-quinones (diphenolase) [1]. Tyrosinase is profoundly involved in melanin synthesis via *O*-hydroxylation of tyrosine into 3,4-dihydroxyphenylalanine (l-DOPA) and oxidation of l-DOPA into dopaquinone, that further transformed into melanin through multiple enzymatic processes [2]. Practically, the biosynthetic pathway of melanin in microorganisms is quite different from humans; in fungi, melanin was mainly synthesized from 1,8-dihydroxynaphthalene (DHN) precursors (DHN-melanin) [3,4] through the polyketide pathway, with maonyl-CoA as the starter and extender units [5]. In humans and few microbes, melanin pigments are synthesized by l-DOPA pathway based on tyrosine as precursors that hydroxylated to l-DOPA, then oxidized into dopaquinone and further into melanin pigment [6]. Melanin is the main pigment in skin, hair, and eyes of human, produced by melanocytes through melanogenesis [7], which is the most important photoprotective factor in response to ultraviolet radiation damage from the sun [8]. The melanogenesis process is initiated with the oxidation of l-tyrosine to dopaquinone (DQ) by tyrosinase and the resulting quinone serves as a substrate for the synthesis of eumelanin and pheomelanin [9]. The synthesized DQ undergoes intramolecular cyclization to produce indoline, leukodopachrome (cyclodopa), and dopachrome [2,9,10]. Dopachrome gradually decomposes to give dihydroxyindole (DHI) and dihydroxyindole-2-carboxylicacid (DHICA) [9]. The biosynthetic mechanisms of melanin in fungi is illustrated in Scheme 1. Melanin is a negatively charged amorphous polymer derived from multiple enzymatic and autoxidative polycondensation of various quinone groups of hydrophobic properties [10]. Melanin pigments retain the ability to deactivate free radicals, peroxides and absorb heavy metals and toxic electrophilic metabolites, thus exhibiting profound antioxidant activity [4,5]. Melanin pigments absorb light in a wide spectrum range enclosing UV, their absorption intensity decreases slowly with increasing light wavelengths, and upon interaction with the UV radiation (240–300 nm), photoionization and subsequent partial destruction of melanins can be observed [5,11].

The theory of stimulating melanogenesis upon overexposure to UV light stated the damage to the DNA of melanocyte, thus over-inducting the melanocyte-stimulating hormones (MSH), that in turn stimulates the tyrosinases activities, multiplying the numbers of melanocytes [6,10,12,13]. The hyper-pigmentation, due to the overactivity of tyrosinase, is usually associated with pathological disorders such as age spots, ephelides, melasma, and senile lentigines [7,8]. Thus, regulating the productivity of tyrosinase is a metabolic challenge to balance the proper melanogenesis and disorders from hyperpigmentation [1,12,13,14].

Tyrosinase is a highly conserved enzyme showing high proximity in molecular structure and catalytic properties among human, vertebrates, plants, and fungi. Thus, using fungal tyrosinase as a model enzyme for evaluating the kinetics of inhibition toward various natural bioactive compounds could a reliable approach due to the feasibility of purification compared to the human enzyme [1,2]. Tyrosinase is a ubiquitous enzyme in most of the microorganisms; it was first identified from *Streptomyces* sp. [15], then *Bacillus megaterium*, *B. thuringiensis*, *Rhizobium* sp., *Pseudomonas maltophilia*, *P. putida Sinorhizobium meliloti*, *Marinomonas mediterranea*, *Thermomicrobium roseum*, and *Ralstonia solanacearum* as reviewed by [12]. Also, tyrosinase was characterized from various fungal species such as *Aspergillus nidulans*, *A. oryzae*, *A. niger*, *Amanita muscaria*, *Trichoderma reesei,* and *Neurospora crassa* as reviewed by [12]. However, the extensive characterization and kinetics of inhibition by different bioactive compounds are scarcely characterized. Thus, the main objective of this study is to purify tyrosinase from different fungal isolates with higher catalytic efficiency toward tyrosine, evaluating their biochemical properties by emphasizing on the kinetics of inhibitions to novel bioactive metabolites.

## 2. Materials and Methods

### 2.1. Screening for the Potent Tyrosinase Producing Fungal Isolates

Forty fungal isolates were selected from our lab stock culture [13,16,17,18,19,20,21,22,23,24], and their potency to grow on l-tyrosine as the sole nitrogen source was determined using modified Czapek’s-Dox agar media with 0.5% tyrosine. The media was centrally inoculated with the experimented fungal plug of 6 days old grown on potato dextrose agar [25], incubated for 5 days at 30 °C. The developed fungal colonies were selected and screened for tyrosinase production by growing on Czapek’s-Dox broth medium of 0.5% tyrosine as the sole nitrogen source. A plug of the developed fungal isolate was inoculated into 50 mL/250 mL Erlenmeyer conical flaks. After incubation for 7 days at 30 °C, the fungal mycelial pellets were collected, and washed by Tris-HCl (pH 7.0, 5 mM). Five grams of the fungal fresh weight were pulverized in liquid nitrogen, dispensing in Tris-HCl (pH 7.0, 5 mM) of 1 mM EDTA, 1 mM PMSF and 1 mM DTT [26,27,28]. The mixture was vortexed for 5 min, then centrifuged at 8000 rpm for 10 min at 4 °C, and the supernatant was used as the crude source for intracellular enzymes.

### 2.2. Tyrosinase Activity and Concentration

The enzyme activity was assessed based on the amount of released 3,4-dihydroxyphenylalanine (l-DOPA) as described by Masamoto et al. [29], with slight modifications. Briefly, the reaction mixture contains 50 mM l-tyrosine in Tris-HCl buffer (10 mM, pH 7.0) and 500 µL enzyme preparation in 1 mL total reaction volume. The reaction mixture was incubated for 30 min at 37 °C.

Blanks of reaction at zero-time, reaction without enzyme and reaction without substrate, were used as baselines. The enzymatic reaction was stopped by 10% TCA, centrifuged at 10,000 rpm for 5 min, the supernatant was used, and the released l-DOPA was measured at wavelength 292 nm, regarding to the different concentrations of authentic l-DOPA (Cat.# 59-92-7). One unit of tyrosinase was expressed by the amount of enzyme releasing µmol l-DOPA per mg enzyme per min. The enzyme protein concentration was measured by Folin’s reagent [30], comparing to a known concentration of bovine serum albumin.

### 2.3. Morphological and Molecular Identification of the Potent Fungal Isolates

The potent tyrosinase producing fungal isolates were identified based on their morphological features according to the identification keys of the genera *Aspergillus* [31], *Penicillium* [32], and *Fusarium* [33]. The morphologically identified fungal isolates were further confirmed based on the sequence analysis of their internal transcribed spacers (ITS) region [23,27,34,35,36]. The fungal genomic DNA was extracted with cetyltrimethyl-ammonium bromide (CTAB) reagent [13]. The fungal mycelia (0.2 g) were pulverized in liquid nitrogen, suspended in 1 mL CTAB extraction buffer (2% CTAB, 2% PVP40, 0.2% 2-mercaptoethanol, 20 mM EDTA, 1.4 M NaCl in 100 mM Tris-HCl (pH 8.0)). The gDNA was used as the template for PCR with primers; ITS4 5′-GGAAGTAAAAGTCGTAACAAGG-3′ and ITS5 5′-TCCTCCGCTTATTGATATGC-3′ using 2× PCR master mixture (*i*-Taq^TM^ Cat. # 25027) according to the manufacturer’s instruction. The PCR amplicons were resolved on 1.5% agarose gel in 1× TBE buffer (Cat. # AM9864, Ambion, Invitrogen™, Carlsbad, CA, USA) normalizing to 1 kb DNA ladder (Cat. # PG10-55D1, Puregene). The amplicons were eluted and sequenced (Applied Biosystems^®^ 3500 and 3500xL Genetic Analyzers, Applied Biosystems, Life Technologies, Foster City, CA, USA,) using the same primer sets. The retrieved sequences were non-redundantly BLAST searched on the NCBI database. For the multiple sequence alignment, the sequences were imported into MEGA 7.0 portal aligned with ClustalW muscle algorithm, and the phylogenetic tree was constructed using the neighbor-joining method [37,38].

### 2.4. Bioprocess Optimization of the Potent Fungal Isolate by Two-Factorial Plackett–Burman Design

The selected physicochemical properties namely peptone, casein, ammonium nitrate, beef extract, phenylalanine, yeast extract, urea, tyrosine, asparagine, glycine, valine, cysteine, methionine, glutamic acid, sucrose, starch, glucose, pH, and incubation time (day) were optimized using two factorial Plackett–Burman design to identify the significant variable tyrosinase production [24,25,39]. The nineteen variables were optimized by Plackett–Burman design. Each variable was represented by high (+) and low (−) levels. The design of Placket–Burman depends on the first-order reaction: *Y* = *β*0 +Σ*βiXi* where, Y is the predicted enzyme activity, Xi is an independent variable, βi is the linear coefficient, and β0 is the model intercept. All the runs were conducted in triplicates and the average of epothilone production was used as the response. After the desired incubation conditions, the fungal cultures were collected, and the intracellular proteins were extracted, and the enzyme activity was determined as described above.

### 2.5. Purification, Molecular Mass, and Subunit Structure of Tyrosinase

The potent tyrosinase-producing fungal isolates were grown on the optimized media for enzyme production following to the factorial design optimization with the surface response methodology. One cultural plug of the potent fungal cultures was inoculated into 50 medium/250 mL Erlenmeyer conical flask with the optimum media, incubated at the desired incubation conditions. The mycelial pellets were collected and washed by sterile potassium phosphate buffer. The fungal pellets (100 g) were pulverized in liquid nitrogen, dispensed in 100 mL extraction buffer Tris-HCl (pH 7.0, 10 mM) with 1 mM PMSF and 1 mM EDTA. The intracellular proteins were extracted as described above. The enzymatic preparation was fractionally concentrated with 20 kDa cut-off dialyzer (20 kDa, 546-00051 Wako Chemicals, USA) against the same buffer, then concentrated by dialysis against polyethylene glycol 6000, and purified by gel-filtration chromatography using Sephadex-G_200_ column [18,22,30,31,36,38]. The column was pre-equilibrated with Tris-HCl buffer (pH 7.0, 20 mM), loaded with the enzyme and eluted by the same buffer. The activity and concentration of tyrosinase were determined as described above. The molecular homogeneity of active enzymatic fractions was assessed by SDS-PAGE, the most active and molecularly homogenous fractions of tyrosinases were collected and concentrated with 10 K ultra-centrifugal membrane and stored at 4 °C for further analysis.

The molecular homogeneity and subunit structure of purified the purified tyrosinase from the potent fungal isolates were checked by SDS-PAGE [40], with slight modifications. The molecular subunit structure of purified enzyme was determined, comparing to authentic protein marker (Puregene, Cat. # PG-PMT2962 315-10 kDa). The entire molecular mass of the purified enzyme was determined by the native-PAGE [41,42,43,44,45,46,47].

### 2.6. Biochemical Properties of the Purified Tyrosinase from the Potent Fungal Isolates

The biochemical properties of the purified tyrosinase from the potent fungal isolates were determined [13,18,20,27,34,35,36,41,42,43,44,45,46,47]. The optimum reaction temperature for tyrosinase activity was determined by incubating the reaction mixture at different temperatures (30–45 °C), then measuring the enzymatic activity by the standard assay. The thermal stability of purified tyrosinases was evaluated by preincubation of the enzyme at different temperatures (20–50 °C), then measuring the residual activity after 30, 60, 180, and 240 min. The thermal kinetic parameters such as half-life time (*T1/2*), and thermal inactivation rate (*kr*) were determined [13,18,27]. The storage stability of the enzyme at 4 °C was determined after 5, 20, 30, 60, and 90 days by the standard assay.

The optimum pH for the purified tyrosinases was assessed using reaction mixture of different pH values (3–10), then measuring the enzymatic activity by the standard assay. The pH stability of purified enzyme was evaluated by pre-incubation of the enzyme at different pH values (5.0–9.0), then measuring the residual enzymatic activity by the standard assay.

### 2.7. Effect of Various Inhibitors and Fungal Extracts

The effect of different inhibitors on the activity of purified tyrosinase from the potent fungal isolates was assessed. The enzymes preparations were desalted by dialysis (Cat # 546-00051, Wako Pure Chem Corp, Osaka, Japan) against 50 mM Tris-HCl buffer (pH 8.0) of 1mM EDTA. Different cations such as Ba^2+^, Fe^3+^, Ca^2+^, Hg^2+^, Al^3+^, Zn^2+^, Na^+^, and Cu^2+^ were added to the enzymes at 1 mM final concentration, after 2 h of incubation at 4 °C, the substrate was added, and enzymatic activities were measured by the standard assay.

### 2.8. Effect of Kojic Acid, and Extracts of Different Endophytic Fungal Isolates

Different endophytic fungal isolates such as *A. flavus*, *A. flavipes*, *A. niger*, *A. ochraceus*, *A. terreus*, and *A. oryzae*, endophytes of *Moringa oleifera* Lam, were used as source of tyrosinase inhibitors [25]. The fungal isolates were grown on potato dextrose broth media and incubated for 10 days; the cultures were the filtered, and the filtrates were extracted by double volume of ethyl acetate and dichloromethane [28,48,49]. The extracts were concentrated by a rotary evaporator and their inhibitory effect to the activity of tyrosinase was evaluated by the standard assay. The enzyme was incubated with different concentrations of each extracts, in addition to kojic acid (5, 20, 50, 100 μg/mL) for 2 h at 30 °C, then amended with the substrate, and the residual activity of the enzyme was determined by the standard assay. The IC_50_ values of kojic acid as well as the fungal extracts were determined from the dose-dependent inhibition curve of different concentrations of the compounds with the activity of tyrosinase by the standard assay [13].

### 2.9. Fractionation and GC-MS Analysis of the Bioactive Fractions

The potent fungal extracts were fractionated by thin layer chromatography using Merck 1 mm (20 × 20 cm) pre-coated silica gel plates (TLC Silica gel 60 F254, Darmstadt, Germany). The plates were developed with the solvent system; dichloromethane and methanol (95:5) [18,35]. The TLC plate was visualized by UV illumination at 254 nm (MinUVIS, Heid., Germany), and the colored spots were scraped-off from the silica, re-dissolved in dichloromethane, vortex for 3 min, centrifuged for 5 min at 5000 rpm to precipitate the silica particles. The eluted fractions were checked for activity against tyrosinase, as described by the standard assay.

The most active fractions against tyrosinase activity was further chemically identified by gas-liquid chromatography analysis. The GC-MS system (Agilent Technologies) was equipped with gas chromatograph (7890B) and mass spectrometer detector (5977A), equipped with HP-5MS column (30 m × 0.25 mm internal diameter and 0.25 μm film thickness [27,28,44,50]. Samples were diluted with hexane, analyses were carried out using helium as the carrier gas at a flow rate of 1.0 mL/min at a split ratio of 1:10, injection volume of 1 µL and the following temperature program: 40 °C for 1 min; rising at 4 °C/min to 150 °C and held for 6 min; rising at 4 °C/min to 210 °C and held for 1 min. The injector and detector were held at 280 °C and 220 °C, respectively. Mass spectra were obtained by electron ionization (EI) at 70 eV; using a spectral range of *m/z* 50–550 and solvent delay 5 min. Identification of different constituents was determined by comparing the spectrum fragmentation pattern with those stored in Wiley and NIST Mass Spectral Library data.

### 2.10. Deposition of the Fungal Isolates

The potent fungal isolates producing tyrosinase, *Aspergillus terreus* and *Penicillium copticola* were deposited into the NCBI database under accession numbers MT000960.1 and MW433880.1, respectively.

### 2.11. Statistical Analyses

All experiments were conducted in biological triplicates, the results were expressed by means ± STDEV. The significance and *F*-tests were calculated by one-way ANOVA with Fisher’s least significant difference post-hoc test.

## 3. Results and Discussion

### 3.1. Screening for the Potent Tyrosinase Producing Fungi

Forty fungal isolates were selected from our lab (Enzymology and Fungal Biotechnology Lab) and their potency to grow on l-tyrosine as the sole nitrogen source was determined using modified Czapek’s-Dox agar media (Data not shown). Among the experimented fungal isolates, nineteen isolates displayed an obvious morphological growth with plausible fluctuations on tyrosine containing modified Czapek’s-Dox media. The tyrosinase productivity was assessed by the standard assay as described in Materials and Methods. Among the tested isolates, the highest tyrosinase productivity was reported for *Penicillium copticola* (0.51 µmol/mg/min), followed by *Aspergillus terreus* (0.423 µmol/mg/min), *Trichoderma viride* (0.41 µmol/mg/min), and *A. ustus* (0.36 µmol/mg/min) (Table 1). While the other fungal isolates display tyrosinase activity between 0.25 and 0.31 µmol/mg/min. The morphological identities of the potent fungal isolates *Aspergillus terreus* and *Penicillium copticola* were confirmed from the molecular authentication based on their ITS sequences. Similar screening paradigm for enzymes production by fungi were reported [27,42]. Coincidently, different tyrosinase producing fungal isolates have been documented [1,2,17].

### 3.2. Molecular Confirmation of the Identity of the Potent Tyrosinase Producing Fungi

**Figure 1:**
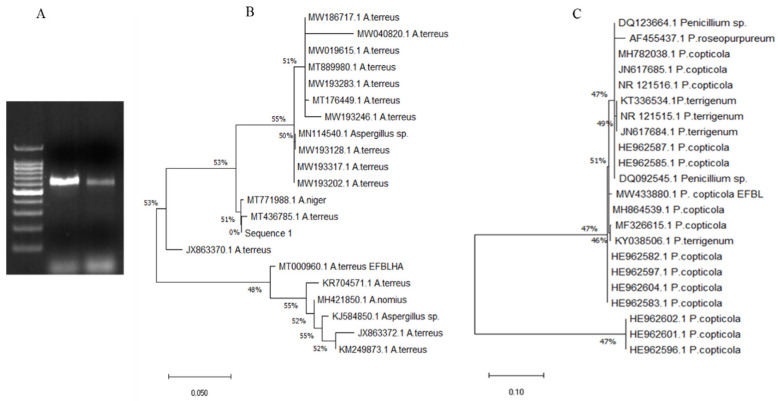
Molecular identification of the potent tyrosinase producing endophytic fungal isolates inhabiting *Moringa oleifera.* (**A**)*,* PCR amplicons of ITS regions of the experimented fungal isolates using genomic DNA as template for PCR with the primers ITS4 and ITS5. Molecular phylogenetic analyses of *Aspergillus terreus* (**B**) and *Penicillium copticola* (**C**) by the maximum likelihood model of MEGA 7.0 package.

The morphological identification of the potent fungal isolates producing tyrosinase was further confirmed based on their ITS region sequences. The genomic fungal DNA was extracted and used as a template for PCR, the sizes of PCR amplicons were found to be around 650 bp (Figure 1). These amplicons were purified, sequenced, and non-redundant BLAST searched in the NCBI database. The fungal isolates were confirmed as *Aspergillus terreus* and *Penicillium copticola* and their ITS sequences were deposited on the NCBI database with accession # MT000960.1 and MW433880.1, respectively. From the alignment profile, applying the Neighbor-Join with the Maximum Composite Likelihood approach, the phylogenetic trees of these sequences have been constructed (Figure 1). The *A. terreus* EFBLHA MT000960.1 displayed a 99% similarity with *A. terreus* database deposited isolates KR704571.1, MH421850.1, JX863372.1, JX863370.1, MT436785.1, MW193202.1, MW193317.1, MW193128.1, MN114540.1, and MW192346.1 with zero *E*-value and 98% query coverage. While, the isolate *Penicillium copticola* EFBL MW433880.1 exhibited 98% similarity with database deposited isolates of *P. copticola* MH864539.1, MF326615.1, KY038506.1, HE962582.1, HE962597.1, HE962583.1, HE962602.1, HE962601.1, HE962596.1, HE962585.1, HE962587.1, NR121516.1, and JN617685.1 with zero *E*-value and 97% query coverage.

**Figure 2:**
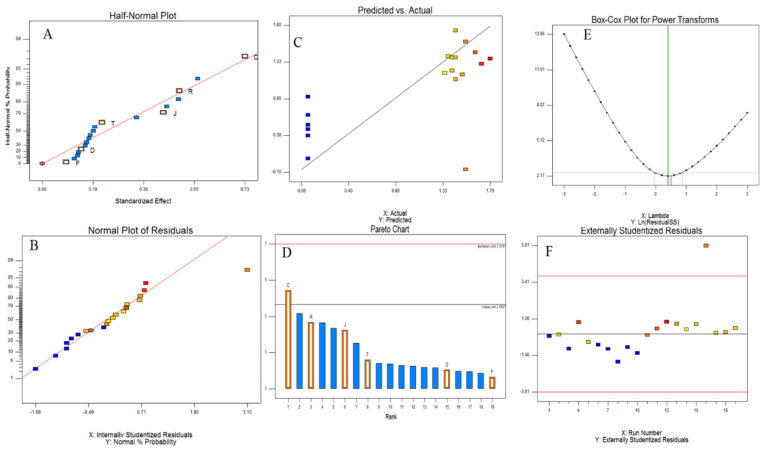
Effects of the different variables on tyrosinase production by *A. terreus* using Plackett–Burman experimental design. The normal probability plots of the variables for tyrosinase production were determined by the first-order polynomial equation (**A**,**B**). Plot of correlation between predicted and actual tyrosinase activity of *A. fumigatus* (**C**,**D**), Pareto chart illustrates the order of significance of each variable. (**E**) Box-Cox power transform. (**F**) Plot of standardized effect with the normal probability. The symbols C refers to the response ammonium nitrate, R refers to glucose, J refers to asparagine, T refers to incubation time, D refers to beef extract, F refers to yeast extract.

Bioprocess optimization of *A. terreus* tyrosinase productivity using Plackett–Burman design Nineteen independent variables were screened on the productivity of tyrosinase by *A. terreus* using Plackett–Burman design (Table 2). The matrix of Plackett–Burman design with the significance of the independent variables affecting tyrosinase productivity by *A. terreus* with the predicted and actual values was shown (Table 3). The fluctuation of predicted yield of tyrosinase as resolved from Plackett–Burman design was obviously wide ranged from 0.065 to 1.7 µmol/mg/min, reflecting the significance of optimization process and efficiency of Plackett–Burman design. The maximum yield of tyrosinase by *A. terreus* was recoded at the run number 13, with the variables concentrations Peptone (1), Casein (−1), Ammonium nitrate (1), Beef extract (−1), Phenylalanine (1), Yeast extract (−1), Urea (−1), Tyrosine (−1), Asparagine (−1), Glycine (1), Valine (1), Cysteine (−1), Methionine (1), Glutamic acid (1), Sucrose (−1), Starch (−1), Glucose (1) at pH 8 and incubation time 10 days. The correlation of tyrosinase productivity and the independent variables was assessed by the multiple-regression statistical analysis of variance (ANOVA) of the experimental design. The effect of the significant variable on the productivity of tyrosinase by *A. terreus* was shown in Figure 2. Pareto Chart reveals the significance of the variables regulating the productivity of tyrosinase by *A. terreus,* as well as, the probability plot of the independent variables, plot of actual and predicted productivity of tyrosinase are shown (Figure 2). The significance of each coefficient was determined from the *p*-value and student’s *t*-test. From the normal probability plot, the points of residuals were arranged near the diagonal line, revealing the independent normal distribution of the variables, suggesting the perfect fitting of the expected epothilone yield with the experimental results. From the Plackett–Burman’s design ANOVA analysis, the constructed model was highly significant as revealed from the values of Fisher’s *F*-test 7.4 and lower probability.

Tyrosinase activity (µmol/mg/min) = 0.98 + 0.36 * ammonium nitrate + 0.07 * beef extract + 0.043 * yeast extract + 0.22 * asparagine + 0.25 * glucose + 0.11 * incubation time.

*p*-value 0.029. The first-order polynomial equation for tyrosinase production by the selected fungal isolate based on Plackett–Burman design is the following:

Thus, upon nutritional optimization by the surface response methodology using the Plackett–Burman design, the yield of tyrosinase by *A. terreus* was increased from 0.43 to 3.2 µmol/mg/min, by about 7.5-folds increment.

### 3.3. Purification, Molecular Subunit Structure of Tyrosinase from A. terreus and P. copticola

**Figure 3:**
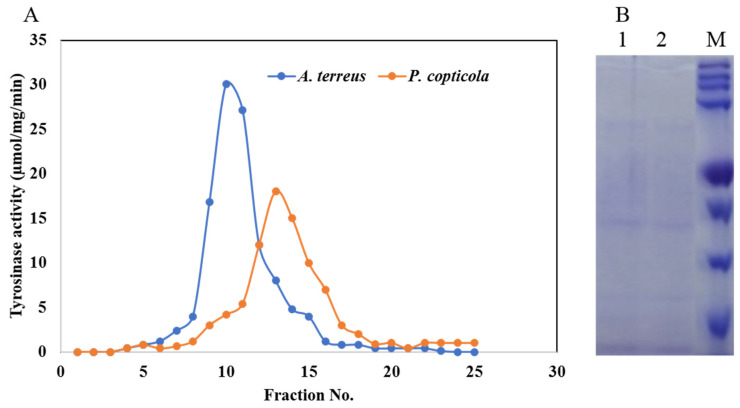
Purification profile of tyrosinase from *A. terreus* and *P. copticola* with different chromatographic approaches. The experimented fungal cultures were incubated at the desired conditions for tyrosinase production, the fungal tissues were pulverized in liquid nitrogen and the intracellular proteins were extracted, used as a source of crude enzyme as described in Materials and Methods. (**A**) The precipitated crude tyrosinases from both fungal isolates were purified by gel filtration chromatography with Sepadex-G-200 column from *A. terreus* and *P. copticola*. The molecular subunit structure of the purified tyrosinase after the last purification step, was checked by the SDS-PAGE (**B**). M, Protein ladder (xxx), 1 *A. terreus* and 2 *P. copticola* purified tyrosinase.

The potent tyrosinase producing fungal isolates “*A. terreus* and *P. copticola*” were grown in nutritionally optimized conditions derived from the Plackett–Burman Design. After incubation of the cultures at standard conditions, the enzyme was extracted, fractionally concentrated by dialysis membrane, precipitated and purified by gel-filtration chromatography. The overall purification profile of tyrosinase from both fungal isolates is summarized as in Table 4. The homogeneity and overall purification steps were monitored by SDS-PAGE analysis. By the last step, the enzyme from *A. terreus* and *P. copticola* was purified by about 13.8- and 11.2-folds, respectively. The specific activity of purified tyrosinase from *A. terreus* (28.1 µmol/mg/min) was increased by 13.8-folds compared to the crude enzyme (2.03 µmol/mg/min), with overall yield of 63.9%. While, the specific activity of purified tyrosinase from *P. copticola* (23.27 µmol/mg/min) was increased by about 11.2-folds, compared to the crude enzyme (2.09 µmol/mg/min), with overall yield of 56.3%; the activity of these fungal tyrosinases being consistent with tyrosinases from *A. oryzae*, *N. crassa,* and *P. sanguineus* [48]. The activity of purified tyrosinase of *A. terreus* and *P. copticola* was strongly higher that the activity of various bacterial tyrosinase especially *Pseudomonas putida* which usually ranged from 0.1 to 12 µmol/mg/min [49,50]. The molecular homogeneity of the most active fractions was checked by SDS-PAGE, and the only most homogenous fractions were gathered and concentrated. The molecular subunit structure of the purified tyrosinase from *A. terreus* and *P. copticola* was around 35 kDa as revealed from the non-denaturing-PAGE (Figure 3). The molecular subunit structure of tyrosinases from experimental fungal isolates was coincident with tyrosinases from *N. crassa* [51], *A. oryzae* [52], and *Pycnoporus sanguineus* [48] that belong to mushroom tyrosinase. There are four structurally different isomers of tyrosinases ranging mainly from monomers to octamers, however, the predominant fungal tyrosinase is mainly a homo-tetrameric enzyme, i.e., four identical subunits of ~35 kDa [53], consistent to with the current results.

### 3.4. Biochemical Properties of the Purified A. terreus and P. copticola Tyrosinase

#### 3.4.1. Reaction Time, Reaction Temperature, Thermal Stability, Reaction pH, and pH Stability

**Figure 4:**
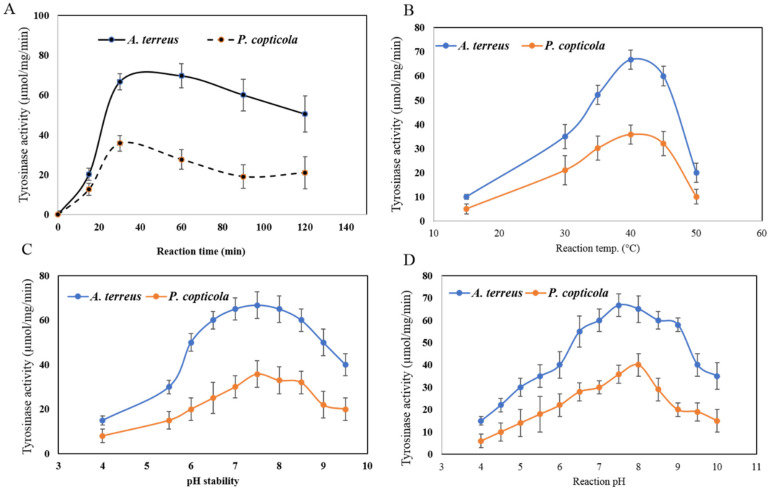
Biochemical properties of the purified tyrosinase from *A. terreus* and *P. copticola.* The effect of reaction time (**A**), reaction temperature (**B**), pH stability (**C**), and reaction pH (**D**) on the purified tyrosinase from both fungal isolates.

The reaction kinetics of the purified tyrosinase from both fungal isolates was evaluated. From the results (Figure 4A), a noticeable exponential increasing of the enzymatic activity was observed with the increasing of incubation time till maximum activity at 30 min at 37 °C followed by a relative stability. The enzyme from both fungal sources exhibit the same catalytic response, revealing the complete proximity on their molecular and tertiary structure, however, the enzyme from *A. terreus* displays a higher catalytic efficiency and turnover number than *P. copticola*. The exponential increasing of the enzymatic activity could be related to the potential probability of the number of substrate molecules to bind adequately with the active sites per enzyme molecule. At the logarithmic phase, a maximum binding of substrate molecules with the enzyme active sites tales place, unlike the oversaturation of the enzyme active sites with the substate after 30 min of incubation. Coincidently, the kinetics of enzymatic activity with the reaction time has been extensively reported for various enzyme [13,20,26,27,28,34].

The effect of reaction temperature on tyrosinase activity from both fungal isolates has been assessed. A gradual increase of the enzymatic activity was reported with the reaction temperature till the maximum value at 37–40 °C, followed by a dramatic decrease in the enzymatic reaction (Figure 4B). The enzymes have the same temperature response pattern, revealing the proximity of the tertiary structure and catalytic efficiency of enzyme from both sources. The highest tyrosinases activity from *A. terreus* and *P. copticola* was 66.7 and 35.8 µmol/mg/min, respectively, at 37 °C. While, at reaction temperature 15 °C, the activity of both the enzymes was reduced by about 80% compared to the optimal activity at 37 °C, suggesting the lower activation energy for substrate to bind with the enzyme active sites. Also, at 50 °C, a dramatic decrease in the enzyme activity by about 85% was seen, which might be due to enzyme denaturation or dissociation of subunits, or tertiary unfolding shielding the surface active sites, as reported frequently for various enzymes in response to higher reaction temperatures [13,16,20,26,27,28,34,41,42]. The maximum activity of the purified tyrosinase at 37 °C, could be a plausible criterion for using this enzyme as a model to the human enzyme, revealing their molecular and catalytic proximity.

The effect reaction pH on the activity of tyrosinases from both fungal sources was evaluated in the pH range 3.5 to 10.0 using different buffers. The highest activity of tyrosinase from *A. terreus* (66.8 µmol/mg/min) and *P. copticola* (40.1 µmol/mg/min) was reported at pH 7.5 (Figure 4C). A strong reduction in the enzymatic activity was reported at lower (pH 4.0) and higher pH (10.0), approximated by 84–95%, compared to the control at pH 7.5. The dramatic reduction in the enzymatic activity at lower and higher reaction pH might be due to denaturation of the enzyme subunits, or changing of the surface charges of the enzyme and their active sites, reducing the binding of substrates with the active sites, or destabilizing the enzyme substrate complex, reducing the overall enzymatic activity [18,38,50,54]. Coincidently, the activity of tyrosinase from both fungal isolates was similar to human tyrosinase and intrinsic pH of melanosome, for melanin biogenesis in human [54].

The pH stability of tyrosinase from both fungal sources was assessed by preincubation at different pH values, then measuring the residual enzymatic activity by the standard assay. From the pH stability pattern (Figure 4D), the enzymes retain their maximum structural stability and catalytic efficiency at pH a range from 7.0 to 8.0 for 2 h. However, the enzymes lose more than 90% of their initial activities at pH 4.0 and pH 10.0. Similar results have been reported for *Trichoderma reesei* [55]. The enzyme from both fungal sources display the same structural and catalytic stabilities in response to different pH values, assuming their structural proximity. Similarly, the human tyrosinase displays the highest pH stability at 7.0 to 8.0 [56]. The dramatic reduction in the tyrosinase activity at lower and higher pH could be due to change on the ionic state of the enzyme, causing dissociation of the subunits, and structural denaturation, releasing the enzyme cofactors [31,36,44]. Taken together, from the biochemical properties of the purified tyrosinase and due to their highly structural and catalytic proximity with human enzyme, it could be used as a model enzyme for studying the kinetics of different inhibitors for melanin biosynthesis for various therapeutic and medical applications.

#### 3.4.2. Effect of Various Cations Inhibitors and Extracted Compounds from Various Endophytic Fungi

**Figure 5:**
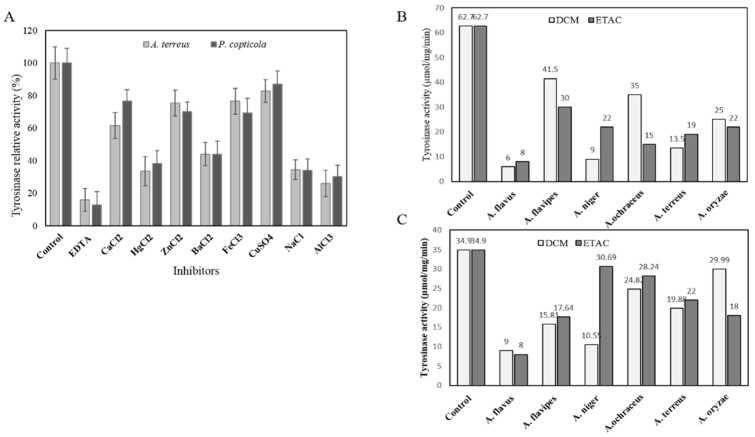
Effect of different metals inhibitors and extracts of different fungal extracts on the purified tyrosinase activity from *A. terreus* and *P. copticola.* The enzyme was preincubated with different cations metals for 2 h, then the residual enzymatic activity was determined by the standard assay. Six fungal isolates (**A**)*. flavus, A. flavipes, A. niger*, *A. ochraceous*, *A. terreus* and *A. oryzae*) were grown for 10 days on PDB media, then the filtrates were extracted with dichloromethane and ethyl acetate. The enzyme was preincubated with the fungal extracts for 2 h, then the residual enzymatic activity from *A. terreus* (**B**) and *P. copticola* (**C**) was measured by the standard assay.

The effect of various metals cations on the activity of the purified tyrosinases has been evaluated. Due to the metalloproteineous identity of human and microbial tyrosinase [57], the purified enzyme was first demetallized by dialysis against EDTA [36,46] then the demetallized enzyme was incorporated with different metals at 1 mM final concentration. The enzymatic activity was measured by the standard assay. Practically, the demetallized enzymes (apoenzymes) retains only about 15% of their initial activities, ensuring the metalloproteinic identities of this enzyme from the fungal origin. From the results (Figure 5), with the incorporation of metals cations such as Ca^+2^, Zn^+2^, Fe^+2^, and Cu^+2^, the enzyme restored about 70% of their original activities compared to the apo-tyrosinase. However, the incorporation of cations Na^+^, Hg^+2^, Al^+2^, Ba^+2^, did not display any positive effect on increasing the enzymatic activity for enzyme from both fungal sources. Restoring the activity of apo-tyrosinase ensures the metalloproteinic identity of this enzyme [36,46]. The relatively similar effects of Ca^+2^, Zn^+2^, Fe^+2^, and Cu^+2^ on the activity of tyrosinase might be due to the proximity of their molecular/stereo-structure, for binding with the cofactor binding sites of enzymes giving a similar effect to the overall catalytic activity of tyrosinase.

The effect of various fungal extracts on the activity of tyrosinase was assessed. Different fungal isolates; *A. flavus*, *A. flavipes*, *A. niger*, *A. ochraceous*, *A. terreus*, and *A. oryzae* were grown on liquid PDB under standard culture conditions. The putative bioactive compounds were extracted with dichloromethane and ethyl acetate, and their activity against tyrosinase was assessed. From the results (Figure 5), the activity of tyrosinase from both fungal isolates was strongly reduced upon using the extracts of *A. flavus* by about 6-8 folds compared to the control enzymes (without inhibitor). However, the other fungal extracts displayed a relatively lower activity toward tyrosinase activity from both fungal isolates, revealing the difference in metabolic pattern of these fungal extracts. The uniqueness of metabolic pattern of each fungal isolate among the same fungal genus and species is a common physiological and metabolic behavior in fungi [58].

#### 3.4.3. Kinetics of Inhibition of Tyrosinase by Kojic Acid and Dichloromethane Extracts of *A. flavus*

**Figure 6:**
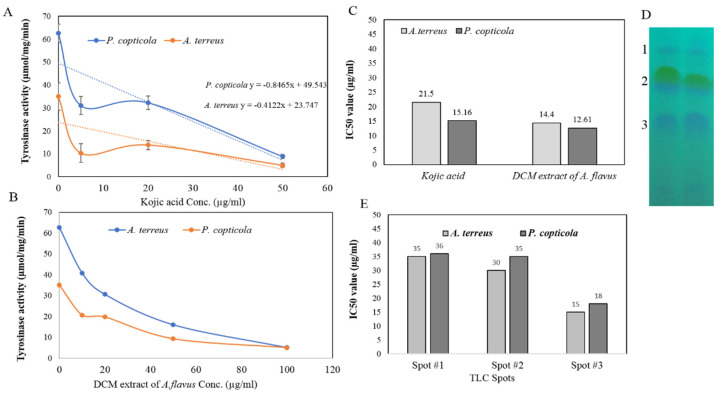
Kinetics of inhibitions of tyrosinases from *A. terreus* and *P. copticola* by kojic acid and DCM extracts of *A. flavus*. Different concentrations of kojic acid (**A**), and *A. flavus* extracts (**B**) were added to the reaction mixture, and the enzyme activity was measured. (**C**) The IC_50_ values of kojic acid and *A. flavus* DCM extract for the enzyme from both fungal isolates. (**D**) TLC chromatogram of the DCM extract of *A. flavus.* (**E**) The IC_50_ values of the three putative spots #1, 2, and 3 for inhibition of tyrosinase of *A. terreus* and *P. copticola*.

The kinetics of inhibition of the tyrosinase by kojic acid and DCM extracts of *A. flavus* were investigated. Kojic acid has been used as the common inhibitor to melanin biosynthesis because of the selective blocking to tyrosinase activity [14,59]. Different concentrations of kojic acid and DCM extracts of *A. flavus* (10–100 µg/mL) were incorporated to the reaction mixture of tyrosinase, and the enzymatic activity was measured by the standard assay. From the reaction kinetics (Figure 6), the activity of tyrosinase was strongly inhibited in a concentration-dependent manner in presence of kojic acid and DCM extracts of *A. flavus*. The initial activity of tyrosinase from both fungal isolates was reduced by about 65% at 40 µg/mL kojic acid compared to control. Similarly, the initial activity of tyrosinase was reduced by about 75% at 50 µg/mL DCM extracts of *A. flavus.* The IC_50_ values of kojic acid and *A. flavus* DCM extracts for inhibiting the activity of tyrosinase from both fungal isolates were calculated (Figure 6C). The IC_50_ values of kojic acid and *A. flavus* DCM extract were 21.5 µg/mL and 14.4 µg/mL for *A. terreus* tyrosinase. While, the IC_50_ values were 15.1 µg/mL and 12.6 µg/mL for kojic acid and DCM extracts of *A. flavus*, respectively. Thus, from the IC_50_ values, the anti-tyrosinase efficiency of DCM extract of *A. flavus* was biologically similar to kojic acid, as common tyrosinase blockers, inhibiting melanin biosynthesis [14,60]. Similar results reporting the production of kojic acid from *A. flavus* with potential activity to inhibit tyrosinases activity can be found elsewhere [27].

Since the DCM extracts of *A. flavus* exhibits a higher anti-tyrosinase activity comparing to kojic acid, thus, further chemical analysis was conducted to resolve the most active components that selectively inhibit tyrosinase activity. The DCM extracts of *A. flavus* was fractionated by TLC as described in Materials and Methods. After development, the TLC plate was visualized by UV-illumination, and three common spots appeared (Figure 6D); these spots were scrapped-off from the plate, eluted and their inhibitory activity to tyrosinase was estimated. The IC_50_ values of the resolved spots were assessed against tyrosinase activity for both fungal isolates. From the results (Figure 6E), the spot number 3 gave the highest inhibitory activity for tyrosinases as revealed from the IC_50_ value that was around 15–18 µg/mL. From the inhibitory activity, the spots # 1 and 2 displayed a slightly lower activity against tyrosinase than fraction # 3. The IC_50_ values of the spots # 1, 2 were approximated 30–35 µg/mL from both fungal isolates. Thus, based on the higher anti-tyrosinase activity, the spot # 3 has been selected for further spectroscopic analysis to resolve the chemical identity of the most active fraction against tyrosinase.

#### 3.4.4. Chemical Identity of the Most Anti-Tyrosinase Active Fraction by FT-IR, H NMR and GC-MS Analyses

**Figure 7:**
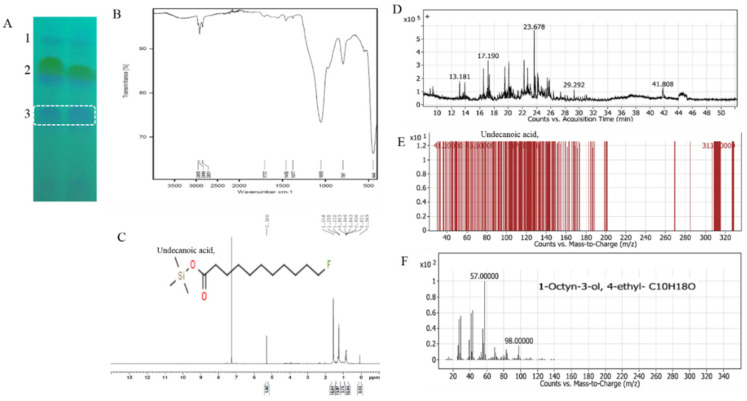
TLC and GC-MS analyses of the potent putative tyrosinase inhibitors (Spot# 3) of DCM extract of *A. flavus.* The potent spots #3 of DCM extract of *A. flavus* was fractionated by TLC, and the visualized spots by UV-illumination were scarped-off and their inhibitory activity for tyrosinase was assayed by the standard assay. TLC chromatogram of the DCM extract of *A. flavus* (**A**), the FT-IR spectra (**B**), and HNMR spectra (**C**) of the putative spots #3. The GC-MS chromatogram of the spots #3 (**D**), the resolving spectra and putative compounds from the LC-MS analyses (**E**,**F**).

The chemical identity of the most active fraction, spot #3, against tyrosinase activity was resolved. The spot # 3 was scrapped-off from the TLC silica, eluted and chemically analyzed by FT-IR, H NMR, and GC-MS analyses [13,25]. From the FT-IR chromatogram (Figure 7B), a slight shifting on the N-H groups from 2359 to 2362 cm and N-C groups from 1315 to 1320 cm was observed, suggesting the deprotonation of NH group and amid bond formation. Presence of hydroxyl groups at 3300 cm^−1^ and CO at 1663 cm^−1^ was found due to the hydrogen bonding, in addition to the epoxy ring at 1200 cm^−1^, while the peaks at 2921, 1623–1485, and 1099 are assigned to the aliphatic CH stretch, ester groups stretch, and aromatic ring stretch, respectively.

From the HNMR spectra, a signal with *δ* at 0.84 ppm for NH_3_ groups was observed (Figure 7). Triplets (4 H, 2CH_2_ at 1.03, 1.05, and 1.29 ppm) for 6 CH_3_ 21, 22, and 27 orientation were observed. Singlet and multiple for 12 protons and 2 hydroxyl (OH) were reported at 2.4 and 4.2, respectively.

From the GC-MS/MS chromatogram of the spot # 3 (Figure 7), two major compounds were resolved namely undecanoic acid, and 1-octyn-3-ol at retention time 23.6 min and 17.19 min, respectively. The arbitrary putative area was 22.1% and 16.2% for undecanoic acid, and 1-octyn-3-ol, respectively. Thus, the main putative major compound with anti-tyrosinase activity has been resolved as undecanoic acid. Interestingly, undecanoic acid has been recognized frequently with its biological activity against pathogenic fungi, bacteria and different microbes [61,62]. However, this is the first report describing the affinity of this compound “Undecanoic acid” to an inhibitor of tyrosinase with comparable activity to kojic acid as commercial melanin biosynthesis inhibitors.

## 4. Conclusions

Tyrosinase productivity has been screened from various filamentous fungal isolates. Among these fungi, *A. terreus* and *P. copticola* were reported as the most potent tyrosinase producers. The overall yield of tyrosinase was maximized upon nutritional optimization bioprocess using the Plackett–Burman design. The enzyme activity from both fungal isolates were increased by about 7.5-folds with the response surface methodology optimization process over the control media. The enzyme was purified, and its various biochemical and catalytic properties were assessed, displaying a noticeable proximity to human tyrosinase, emphasizing the usage of this enzyme as a model enzyme to study the mechanistics and kinetics of inhibition in response to various compounds. Targeting of the human tyrosinase is the main therapeutic approach to limit the over biogenesis of melanin and its hazardous derivatives, thus controlling the various pathogenetic disorders. The activity of the purified tyrosinase was strongly inhibited by kojic acid and DCM extracts with IC_50_ values, 15.1 and 12.6 1 µg/mL, respectively. From the spectroscopic analysis, the chemical components of the most active compounds toward tyrosinase was resolved as undecanoic acid. Further studies are ongoing to unravel the effect of this potent tyrosinase inhibitor in vivo to block the melanin synthesis in fungi and human, that could be a novel drug to various diseases associated with hyperpigmentation by melanin.

## Figures and Tables

**Table 1:** Screening for tyrosinase production by endophytic fungi of Moringa oleifera.

No.	Fungal Isolate	Specific Activity (μmol/mg/min)
1	*Trichoderma* sp.	0.405
2	*Aspergillus terreus*	0.422
3	*Penicillium lilacinum*	0.23
4	*Pestalotia* sp.	0.24
5	*Alternaria alternata* 1	0.17
6	*Alternaria alternata* 2	0.177
7	*penicillium monoverticillate*	0.152
8	*Penicillium citrinum* 1	0.112
9	*penicillium asymmetrica*	0.325
10	*Chaetomium* sp.	0.295
11	*penicillium divericata*	0.245
12	*Aspergillus ornatus*	0.312
13	*Humicola* sp.	0.185
14	*Aspergillus awamori*	0.322
15	*Penicillium citrinum* 2	0.232
16	*Fusarium* sp.	0.26
17	*Aspergillus glaucus*	0.24
18	*Aspergillus ustus*	0.36
19	*Penicillium copticola*	0.507

**Table 2:** Experimental independent variables at two levels for tyrosinase production by *Aspergillus terreus* and *Penicillium copticola* using Plackett–Burman design.

	Variables	Level	
		−1	1
X1	Peptone (g/L)	2	5
X2	Casein (g/L)	2	5
X3	Ammonium nitrate (g/L)	2	5
X4	Beef extract (g/L)	2	5
X5	Phenylalanine (g/L)	2	5
X6	Yeast extract (g/L)	2	5
X7	Urea (g/L)	3	6
X8	Tyrosine (g/L)	2	5
X9	Asparagine (g/L)	2	5
X10	Glycine (g/L)	2	5
X11	Valine (g/L)	2	5
X12	Cysteine (g/L)	2	5
X13	Methionine (g/L)	2	5
X14	Glutamic acid (g/L)	2	5
X15	Sucrose (g/L)	2	6
X16	Starch (g/L)	1	4
X17	Glucose (g/L)	2	8
X18	pH	5	8
X19	Incubation time (day)	5	10

**Table 3:** Twenty-trial Plackett–Burman experimental design for evaluation of fifteen independent variables with coded values and tyrosinase activity by *A. terreus*. The “−1′′ sign correspond to the minimum value and the “+1′′ sign correspond to the maximum value of the input parameter range.

Run	X1	X2	X3	X4	X5	X6	X7	X8	X9	X10	X11	X12	X13	X14	X15	X16	X17	X18	X19	Actual Activity	Predicted Activity	Residuals
1	−1	−1	−1	1	1	−1	1	1	−1	−1	1	1	1	1	−1	1	−1	1	−1	0.008	0.075	−0.075
2	−1	1	1	−1	−1	1	1	1	1	−1	1	−1	1	−1	−1	−1	−1	1	1	1.9	1.39	0.51
3	1	1	−1	1	1	−1	−1	1	1	1	1	−1	1	−1	1	−1	−1	−1	−1	0.07	0.9	0
4	1	−1	−1	1	1	1	1	−1	1	−1	1	−1	−1	−1	−1	1	1	−1	1	2.9	1.302	1.598
5	−1	1	1	1	1	−1	1	−1	1	−1	−1	−1	−1	1	1	−1	1	1	−1	2.1	1.73	0.37
6	1	1	−1	1	−1	1	−1	−1	−1	−1	1	1	−1	1	1	−1	−1	1	1	0.09	0.375	−0.375
7	1	1	−1	−1	1	1	1	1	−1	1	−1	1	−1	−1	−1	−1	1	1	−1	0.03	0.513	−0.513
8	1	1	1	−1	1	−1	1	−1	−1	−1	−1	1	1	−1	1	1	−1	−1	1	0.09	0.87	−0.87
9	1	−1	1	−1	−1	1	1	−1	1	1	−1	−1	1	1	1	1	−1	1	−1	0.1	0.455	−0.455
10	− 1	1	−1	−1	−1	−1	1	1	−1	1	1	−1	−1	1	1	1	1	−1	1	0.1	0.641	−0.641
11	1	−1	1	−1	−1	−1	−1	1	1	−1	1	1	−1	−1	1	1	1	1	−1	2.4	1.59	0.81
12	1	−1	1	1	−1	−1	1	1	1	1	−1	1	−1	1	−1	−1	−1	−1	1	2.7	1.451	1.249
13	1	−1	1	−1	1	−1	−1	−1	−1	1	1	−1	1	1	−1	−1	1	1	1	3.2	1.369	1.831
14	−1	−1	−1	−1	1	1	−1	1	1	−1	−1	1	1	1	1	−1	1	−1	1	2.3	1.162	1.138
15	−1	1	1	−1	1	1	−1	−1	1	1	1	1	−1	1	−1	1	−1	−1	−1	1.8	1.18	0.62
16	−1	−1	1	1	1	1	−1	1	−1	1	−1	−1	−1	−1	1	1	−1	1	1	2.1	1.103	0.997
17	−1	−1	−1	−1	−1	−1	−1	−1	−1	−1	−1	−1	−1	−1	−1	−1	−1	−1	−1	2.4	0.065	2.335
18	−1	−1	1	1	−1	1	1	−1	−1	1	1	1	1	−1	1	−1	1	−1	−1	2	1.38	0.62
19	1	1	1	1	−1	1	−1	1	−1	−1	−1	−1	1	1	−1	1	1	−1	−1	2.1	1.381	0.719
20	−1	1	−1	1	−1	−1	−1	−1	1	1	−1	1	1	−1	−1	1	1	1	1	2	1.21	0.79

**Table 4:** Overall purification profile of TYR from *Aspergillus terreus* and *Penicillium copticola.*

Step	*Aspergillus terreus*					*Penicillium copticola*				
	Total Activity (µmol/min)	Total Protein (mg)	Specific Activity (µmol/mg/min)	Fold	Yield (%)	Total Activity (µmol/min)	Total Protein (mg)	Specific Activity (µmol/mg/min)	Fold	Yield (%)
Crude enzyme	188.44	92.79	2.03	1	100	178.69	105.69	2.09	1	100
Acetone precipitate	147.96	8.94	16.7	8.19	83.83	152	14.16	10.73	5.14	85.1
Sephadex G_200_	120.46	4.32	28.1	13.8	63.93	100.7	4.31	23.37	11.2	56.3

## Data Availability

All the data are included on this paper.

## Sample Availability

Samples of the compounds are available from the authors.
